# Expect the Unexpected Enrichment of “Hidden Proteome” of Seeds and Tubers by Depletion of Storage Proteins

**DOI:** 10.3389/fpls.2016.00761

**Published:** 2016-06-01

**Authors:** Ravi Gupta, Cheol W. Min, Yiming Wang, Yong C. Kim, Ganesh K. Agrawal, Randeep Rakwal, Sun T. Kim

**Affiliations:** ^1^Department of Plant Bioscience, Life and Industry Convergence Research Institute, Pusan National UniversityMiryang, South Korea; ^2^Department of Plant Microbe Interactions, Max Planck Institute for Plant Breeding ResearchCologne, Germany; ^3^Research Laboratory for Biotechnology and BiochemistryKathmandu, Nepal; ^4^Global Research Arch for Developing Education, Academy Pvt. Ltd.Birgunj, Nepal; ^5^Faculty of Health and Sport Sciences and Tsukuba International Academy for Sport Studies, University of TsukubaIbaraki, Japan

**Keywords:** hidden proteome, low-abundance proteins, high-abundance proteins, storage proteins, proteome coverage, depletion methods, plant

## Abstract

Dynamic resolution of seed and tuber protein samples is highly limited due to the presence of high-abundance storage proteins (SPs). These proteins inevitably obscure the low-abundance proteins (LAPs) impeding their identification and characterization. To facilitate the detection of LAPs, several methods have been developed during the past decade, enriching the proteome with extreme proteins. Most of these methods, if not all, are based on the specific removal of SPs which ultimately magnify the proteome coverage. In this mini-review, we summarize the available methods that have been developed over the years for the enrichment of LAPs either from seeds or tubers. Incorporation of these methods during the protein extraction step will be helpful in understanding the seed/tuber biology in greater detail.

## Introduction

The possibility of identifying the whole set of proteins present in a sample, was first proposed over two decades ago when the term “Proteomics” was coined by an Australian Scientist “Marc Wilkins” (Wilkins et al., 1996). Since then, the field of proteomics has flourished at a fast pace and several advancement(s) in the original methods/technologies have been made, which made the proteomic technologies more autonomous, high-throughput and reliable (Sanchez-Lucas et al., 2016). However, the detection and identification of low-abundance proteins (LAPs) have always been challenging due to limitations in the protein separation technologies, and thus, identification of a whole set of proteins in a given sample remains one of the prime goals for the plant proteomers (Righetti and Boschetti, 2016). Presence of high-abundance proteins (HAPs) in the plants tissues, like RuBisCO (ribulose-1,5-bisphosphate carboxylase/oxygenase) in green leaves and various storage proteins (SPs) in seeds and tubers, is one of the major barriers, hampering the detection of LAPs (Gupta et al., 2015).

SPs are accumulated in seeds and tubers during their development and comprise a high proportion (upto 80%) of the total seed/tuber proteins (Shewry, 1995). A major function of these SPs is to act as a source of carbon and nitrogen during the seed and tuber germination, however, some of these proteins also possess enzymatic activities and exhibit beneficial health effects (Shewry, 1995) (Agrawal and Rakwal, 2012). Glycinin and β-conglycinin in soybean seeds (Natarajan, 2014), vicilins in maize seeds (Xiong et al., 2014), sporamin in sweet potato tubers (Lee et al., 2015), patatin in potato tubers (Lee et al., 2015), and dioscorin in yam tubers (Xue et al., 2015), are some of the common examples of SPs. The seed SPs have been classified into five classes on the basis of their solubility in water (albumins), dilute saline solutions (globulins), alcohol-water mixtures (prolamins), and dilute alkali/acid (glutelins) (Shewry, 1995) and (Miernyk and Johnston, 2012). Albumins are present in all the seeds, prolines and glutelins in monocotyledonous seeds and globulins accumulate majorly in the dicotyledonous seeds (Shewry, 1995) and (Miernyk and Johnston, 2012). In case of tuber SPs, no such classification has yet been given, probably because of lesser similarities in their biological properties, indicating independent evolution of these proteins in different species (Shewry, 2003).

To understand the seed and tuber biology, several proteomics studies have been conducted in recent years, however, most of those ended up majorly with the identification of SPs (Agrawal and Rakwal, 2012). Key regulatory proteins are still not being identified because of their low abundance and therefore, some of the basic biological questions related to the seed/tuber development, dormancy, germination, aging and accumulation of metabolites, among others, are yet to be answered. The main reason behind this limitation, as stated before, is the presence of SPs that inevitably obscure these inconspicuous signaling or regulatory proteins (Gupta et al., 2015). Therefore, enrichment of LAPs is prerequisite to address these biological questions.

**FIGURE 1:**
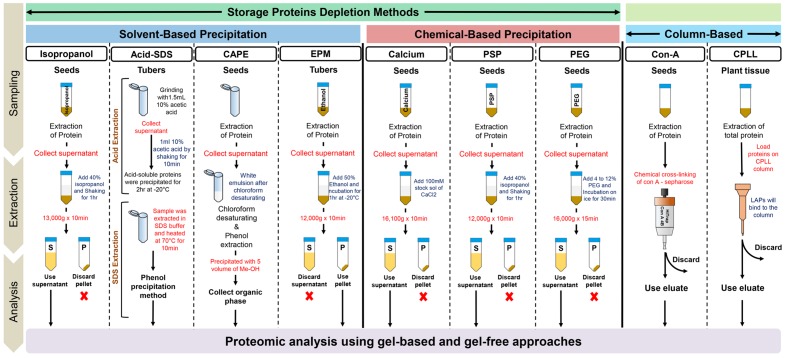
**A summary of the workflows developed for the enrichment of low-abundance proteins (LAPs) in seeds and tuber samples using HAPs depletion and CPLL methods.** Details of these techniques are mentioned in the text and in the cited references.

The idea of identifying the LAPs has always been a hot area of research and this is why several laboratories across the globe are working on the development of methods for enrichment of LAPs (Gupta et al., 2015) (Righetti and Boschetti, 2016). As the major problem in seeds and tubers is the presence of SPs, the logical view is to remove these SPs from the total proteins in order to enrich these LAPs. Therefore, most of the developed methods are based on the specific depletion of SPs as summarized below, and shown in **Figure 1**.

## Methods Available to Analyze the “Hidden Proteome”

### Solvent Based Precipitation

#### Isopropanol Method

Initially, an isopropanol method was developed to deplete glycinin and β-conglycinin subunits from total soybean seed proteins. Authors used 10–80% isopropanol in water to preferentially deplete the seed SPs (Natarajan et al., 2009). Of these concentrations tested, 30–60% isopropanol showed sufficient depletion of both glycinin and β-conglycinin subunits. Further resolution of isopropanol extracted proteins showed that 40% isopropanol had the maximum number of LAP spots as compared to other concentrations (30, 50, and 60%) (Natarajan et al., 2009). Recently, it was shown that ultrasonic treatment during isopropanol extraction increases the amount of LAPs. A 26.42% increase in the LAPs was observed when soybean seed proteins were isolated with 50% isopropanol along with the ultrasonic (400 W) treatment for 10 min (Liu et al., 2016).

#### Acid-SDS Based Extraction

The acid-SDS based extraction method was developed specifically to remove SPs from *Pinellia ternata* tubers, which are widely used in Chinese traditional medicines (Wu et al., 2012). Here, the property of differential solubility of SPs in acids was exploited to remove the most abundant tuber proteins. This method involves extraction of SPs in 10% acetic acid followed by extraction of soluble proteins in SDS-based buffer (0.5 M Tris-HCl, pH 8.8, 2% SDS, and 20 mM DTT). Initially, different concentrations of acetic acid (1, 5, 10, 30, and 60%) were tested of which 10% was found as the optimal concentration for the depletion of major SP of 25 kDa. After acetic acid extraction/precipitation, the pellet thus obtained, was washed twice with chilled acetone to remove the residual acetic acid and then solubilized in a SDS-based buffer. Proteins from the SDS buffer were recovered using phenol-methanolic ammonium acetate precipitation where samples were mixed with an equal volume of phenol. After centrifugation, the lower phenol phase was mixed with five volumes of 0.1 M ammonium acetate in methanol and incubated at -20°C for 2 h to precipitate the proteins. The pellet thus obtained was dissolved in either SDS-PAGE loading buffer or 2D rehydration buffer and directly loaded onto the gels. The 1D and 2D gel profiles showed that 25 kDa SP was almost removed while the 11 kDa SP was enriched along with the LAPs in the pellet-fraction proteins, probably because of its lesser solubility in the acids. This method is unable to remove the acid insoluble SPs from the LAPs, which is one of the drawbacks of this protocol (Wu et al., 2012).

#### Chloroform-assisted Phenol Extraction (CAPE)

Chloroform-assisted Phenol Extraction (CAPE) method was developed to deplete the vicilins, major SPs in maize embryos (Xiong et al., 2014). This method involves extraction of seed proteins first in aqueous buffer [0.25 M Tris-HCl (pH 7.5), 1% SDS, 14 mM DTT and a cocktail of protease inhibitors] followed by denaturation of proteins by chloroform [1:1 (v:v) of extract:chloroform, shaking for 10 min] and finally extraction of proteins using the phenol-methanolic ammonium acetate precipitation method. Post-CAPE, the 2D gels clearly showed the removal of vicilins from the total maize embryo proteins. MS/MS identification of the 17 depleted spots, confirmed those to be the vicilins, further indicated the efficacy of the CAPE in selective depletion of SPs from maize seeds. Moreover, the application of this method was extended in soybean where the depletion of glycinin and β-conglycinin subunits was shown following this protocol (Xiong et al., 2014).

#### Ethanol Precipitation Method (EPM)

Ethanol precipitation method was developed to fractionate the sporamin and patatin, major SPs in the sweet potato and potato tubers, respectively (Lee et al., 2015). This method involves extraction of total tuber proteins in aqueous buffer [0.5 M Tris-HCl (pH 8.3), 2% v/v NP-40, 20 mM MgCl_2_ and 2% v/v β-mercaptoethanol] followed by incubation of total protein extract with 50% ethanol for 1 h at -20°C. Proteins from ethanol-pellet (EP) and -supernatant (ES) fractions, obtained after centrifugation, were isolated using the phenol precipitation method. The 1D and 2D gel profiles clearly showed a dose-dependent fractionation of SPs in the ES fraction and concurrently enrichment of LAPs in the EP fractions. Out of the different concentrations of ethanol tested (20–80%), 50% showed best results in terms of enrichment of LAPs in the EP fraction. A recent study used EPM to compare the anthocyanin biosynthesis in the tuberous roots of yellow and purple sweet potato cultivars (Wang et al., 2016). Increased abundance of starch phosphorylase and phosphoglucomutase was observed in purple cultivar, which indicated that starch degradation might provide higher substrates for anthocyanin biosynthesis in purple-colored as compared to the yellow-colored sweet potato cultivar (Wang et al., 2016). This study further supports the reproducibility and applicability of EPM for comparative proteome analysis.

### Chemical Based Precipitation

#### Calcium Method

The same research group that developed the isopropanol method, also developed a calcium-based method to deplete SPs of soybean seeds (Krishnan et al., 2009). However, the effect of this method on enrichment of LAPs was more pronounced than the previously reported isopropanol method and is applicable to the seeds of many other plants (**Table 1**). In this protocol, seed proteins were first isolated in an aqueous buffer (20 mM Tris-HCl, pH 6.8, containing protease inhibitor cocktail) and then incubated with 10 mM of CaCl_2_ for 10 min with constant shaking followed by centrifugation. The 1D gel profile clearly showed the depletion of approximately 10 bands corresponding to the HAPs. Resolution of calcium-fractionated protein samples on 2-DE and difference in-gel electrophoresis (DIGE) further showed removal of 87 ± 4% of the HAPs, pronouncing the detection of 541 LAP spots. Phosphoprotein staining of the calcium-fractionated proteins on 2-D gels led to the detection of 63 new phosphorylated spots of which detection of 15 spots was enhanced in the calcium-fractionated samples, suggesting the depletion of SPs can be fruitful in the phosphoproteome analysis as well (Krishnan et al., 2009). Recently, the calcium precipitation method was utilized to investigate the Lys-N^𝜀^-acetylome of developing soybean seeds, further confirming the applicability of this method for post-translational modification analysis (Smith-Hammond et al., 2014).

**Table 1:** Comparative analysis of the available methods for the enrichment of low-abundance proteins (LAPs) from seeds and tubers.

S. No.	Method	Sample	Applicability	Reproducibility	Efficacy	Comparative Proteomics	Reference
1	Isopropanol	Seed	Soybean	N.Q	2D gel of calcium precipitated fraction showed complete removal of *β*-conglycinin spots, glycinin spots still observed. 107 spots including several LAPs were identified by MALDI-TOF-MS and MS/MS	N.D	Natarajan et al., 2009
2	Acid-SDS based extraction	Tuber	*Pinellia ternata*	N.Q	Of the two, 25 kDa SP was removed while 11 kDa SP was enriched along with the LAPs following this protocol, as observed on the 2D gels. Using MS/MS, 9 spots corresponding to HAPs, were identified as lectin isoforms	N.D	Wu et al., 2012
3	Chloroform-assisted phenol extraction (CAPE)	Seed	Maize, soybean and pea (both dicots and monocots)	N.Q	2D gel of CAPE prepared samples showed 17 spots were selectively removed or newly detected and 12 spots were enriched. MS/MS identification confirmed depleted spots to be vicilins	N.D	Xiong et al., 2014
4	Ethanol precipitation method (EPM)	Tuber	Sweet potato and potato	N.Q	2D gel of pellet fraction of sweet potato showed 158 more spots than total; 35 LAPs were identified using MALDI-TOF/TOF	Yes	Lee et al., 2015
5	Calcium method	Seed	Soybean, peanut, bean, pea, alfalfa, vetch, lupin, trefoil and American potato	N.Q	Enhanced detection of 541 spots was observed on 2D and DIGE gels (volume increase of > 50%) of which 197 were enriched more than 2.5 fold after calcium precipitation in soybean; some of these were identified by MALDI-TOF MS	Yes	Krishnan et al., 2009; Smith-Hammond et al., 2014
6	Protamine sulfate precipitation (PSP) method	Seed	Soybean, broad bean, pea, wild soybean, and peanut	CV < 12% (approximately 88% reproducibility)	2D gels showed 722 and 502 more spots in peanut and soybean, respectively, after PSP fractionation; 14 enriched spots were identified by MALDI-TOF/TOF	Yes	Kim et al., 2015; Min et al., 2015
7	Polyethylene glycol (PEG) method	Seed	Lettuce	N.Q	133 more spots were observed on the 2D gels after 8% (w/v) PEG treatment; 108 enriched spots were identified by MALDI-TOF/TOF	Yes	Wang et al., 2015
8	Con-A affinity chromatography	Seed	Soybean	N.Q	Significant removal of *β*-conglycinin and other glycosylated proteins on 1D and 2D gels	N.D	Miernyk and Johnston, 2006
9	Combinatorial peptide ligand library (CPLL)	Seed	Olive	N.Q in case of seeds. In case of *Arabidopsis* leaf proteome, CV values were 19–20%	31 more proteins were identified by MS/MS following CPLL in olive seeds	N.D for seeds/tubers	Esteve et al., 2012; Fröhlich et al., 2012

*N.Q, not quantified; N.D, not determined.*

#### Protamine Sulfate Precipitation (PSP) Method

Protamine sulfate precipitation (PSP) method was first developed to precipitate the RuBisCO protein from the leaves of green plants where it was shown that addition of 0.1% protamine sulfate (PS) specifically depletes the RuBisCO in pellet fraction (Kim et al., 2013) (Gupta and Kim, 2015). The application of this method was extended to seed proteomics where it was shown that incubation of seed protein extract with 0.05% PS significantly depleted the SPs of soybean, broad bean, pea, and wild soybean in pellet fraction. In the case of peanut, 0.1% PS was required to differentially fractionate the SPs. Briefly, the proteins were first isolated in the Tris-Mg/NP-40 extraction buffer [0.5 M Tris-HCl (pH 8.3), 2% v/v NP-40, 20 mM MgCl_2_] followed by incubation with required concentration of PS (0.05–0.1%) for 30 min on ice. Finally, supernatant was clarified by centrifugation and PS-supernatant was used as LAPs rich fraction as SPs depleted into the pellet fraction (Kim et al., 2015). Recently, the PSP method was used for the comparative analysis of soybean seeds differing in total protein and oil contents, highlighting the efficacy of PSP method in comparative proteome analysis (Min et al., 2015).

#### Polyethylene Glycol (PEG) Method

Similar to the PSP method, PEG method was initially developed to remove the RuBisCO protein from the rice leaves (Kim et al., 2001). Recently, Wang et al. (2015) found that sequential fractionation of lettuce seed proteins with increasing percentage of PEG showed significant depletion of SPs. Using three different concentrations (4, 8, and 12%), authors found 8% as an effective PEG concentration. Addition of 4% PEG led to the depletion of approximately 55% of total proteins, majority of which were identified as SPs. Moreover, addition of 8% PEG in the supernatant fraction further precipitated the SPs in the pellet fraction (Wang et al., 2015). A total of 133 more spots were visualized in the 2D gel of 8% PEG fractionated sample as compared to total seed sample, indicating the potency of PEG method in the enrichment of LAPs. Using the PEG fractionation method, authors showed the involvement of mevalonate pathway in the germination and thermo-inhibition of lettuce seeds.

### Immuno-Affinity Based Methods

#### Concanavalin-A/Lectin Affinity Chromatography

Many of the SPs, including β-conglycinin of soybean seed and 25 kDa SP of *P. ternata* tubers, are *N*-glycosylated in nature. Therefore, an attempt was also made to use lectin affinity chromatography for the removal of β-conglycinin and other glycosylated proteins from the total soybean seed proteins (Miernyk and Johnston, 2006). Total seed proteins were isolated using the phenol-methanolic ammonium acetate precipitation method and then dissolved in 8 M urea, 10% glycerol, and 0.5% (v/v) Triton X-100. Isolated proteins were loaded onto a Con A-Sepharose column and after washing off the unbound proteins, glycosylated proteins were eluted using 400 mM α-methyl mannoside. The 1D and 2D gel profiles showed significant removal of β-conglycinin and a 26 kDa polypeptide from the unbound proteins. However, one of the major demerits of this protocol was that some of the low abundant glycosylated proteins can also be lost along with abundant glycosylated proteins while other non-glycosylated abundant proteins can be eluted out in flow-through fraction.

#### Combinatorial Peptide Ligand Library (CPLL)

Combinatorial Peptide Ligand Library (CPLL) is one of the most promising technologies for the enrichment of the hidden proteome (Boschetti and Righetti, 2013) and (Righetti and Boschetti, 2016). CPLL has the potential to reduce the concentration of a wide range of HAPs and is compatible to any sample/tissue type. CPLL columns consist of several million hexapeptides that have been generated using 16 different amino acids. These hexapeptides are designed in such a way that these can recognize almost all the proteins present in a sample (Righetti and Boschetti, 2016). When the total protein extract is loaded onto a CPLL column, these proteins bind with their hexapeptide partner beads. HAPs saturate their partner beads first and thus their major fraction remain unbound which is finally washed out as the flow through. On the other hand, when additional protein extract is loaded onto a CPLL column, LAPs keep on binding with their partner beads and thus get enriched, which can then be analyzed by downstream gel-based or gel-free proteomic approaches (Righetti and Boschetti, 2016). Esteve and co-workers utilized the CPLL technology for analyzing the olive seed proteome (Esteve et al., 2012). Following CPLL, 31 more seed proteins including SPs, oleosins and histones, were identified, indicating the efficacy and applicability of CPLL technique in seed proteomics.

## Conclusion

Proteomics has come a long way since its introduction over two decades ago. However, in-depth analysis of seed/tuber protein samples is still required to address some of the basic biological questions related to the seed/tuber development, dormancy, germination, aging, and accumulation of metabolites, among others. Presence of HAPs is one of the major problems that hamper deep analysis of seed and tuber proteomes. Currently, there are a number of methods available, which can be effectively used for the selective depletion of HAPs and enrichment of low-abundance seed/tuber proteins. These methods involve extraction of total seed/tuber proteins in aqueous buffer followed by removal of HAPs. However, all of these methods have their own merits and de-merits (**Table 1**). Depletion methods are rapid, simple and cost-effective but there is always a risk of losing some non-specific LAPs that might co-precipitate along with the HAPs. These LAPs may be of potential interest and their depletion can result in loss of biological information. Therefore, while using these depletion or conventional methods, it is better to use both HAP- and LAP-enriched fractions for proteome analysis, wherever applicable. Moreover, not many of these methods have been tested for the comparative proteome analysis, thus limiting their wide acceptability. Furthermore, reproducibility of these methods is the biggest question as quantitative estimation of the reproducibility is missing for most of the methods (**Table 1**). On the other hand, the immuno-affinity-based methods are highly specific and can be used to enrich the multiple or single protein of interest. If the antibodies are available against the SPs, these can be effectively removed using affinity chromatography to enrich the LAPs. In case where antibodies are not available against the target protein(s), CPLL could be a good choice. However, similar to the depletion methods, affinity-based methods also have limitations. While analyzing the *Arabidopsis* leaf and pumpkin phloem proteins Fröhlich et al. (2012) found that the efficacy of the CPLL was dependent on the protocol used and 22% of the protein loss was observed after CPLL treatment even after the use of an optimized protocol. Authors also claimed that the CPLL technology did not extend, rather shift the detectable proteome and significant increase in the detected proteome was achieved only when results of both crude extracts and CPLL eluates were combined (Fröhlich et al., 2012). Therefore, similar to the depletion methods, it is advisable to use both crude extracts and CPLL eluates for the proteome analysis.

Taken together, there is no “universal protocol” available for the depletion of SPs; and successful enrichment of LAPs depend on the tissue, species, objectives, and conditions of analysis. Future efforts should be given to develop a “golden protocol” that can be effectively used for the enrichment of LAPs from wide range of seeds and tubers samples.

## Author Contributions

All authors listed, have made substantial, direct and intellectual contribution to the work, and approved it for publication.

## Conflict of Interest Statement

The authors declare that the research was conducted in the absence of any commercial or financial relationships that could be construed as a potential conflict of interest.
